# Poor Self-Rated Health Influences Hospital Service Use in Hospitalized Inpatients With Chronic Conditions in Taiwan

**DOI:** 10.1097/MD.0000000000001477

**Published:** 2015-09-11

**Authors:** Vivian Isaac, Craig S. McLachlan, Bernhard T. Baune, Chun-Ta Huang, Chia-Yi Wu

**Affiliations:** From the Rural Clinical School, University of New South Wales, Sydney, Australia (VI, CSM); Department of Psychiatry, School of Medicine, University of Adelaide, Adelaide, Australia (BTB); Departments of Internal Medicine and Traumatology, National Taiwan University Hospital, Taipei, Taiwan (C-TH); and Department of Nursing, College of Medicine, National Taiwan University, Taipei, Taiwan (C-YW).

## Abstract

Our aim was to investigate the association between self-rated health (SRH) and use of hospital services (ie, medical outpatient department, emergency department, and general ward. admissions). Cross-sectional study data were collected from 230 consecutive patients admitted to medical departments of a 2000-bed academic medical center in Taiwan using standardized operating procedures for data collection of SRH (ie, a single-item question inquiring overall perceived health status), medical disorders, depressive symptoms, and combined service utilization over a 1-year period (ie, number of visits to outpatient department, number of visits to emergency department, and number of hospitalizations). Electronic medical records were retrieved, with self-reported external medical visits added to in-hospital frequencies of service use to provide better estimation of health service utilization. Fifty-two percent of study patients rated their health as poor or very poor. Poor SRH was associated with more visits to medical outpatient department, emergency department, and hospital admission. Multivariate logistic regression demonstrated an independent association between poor SRH and services utilization after adjustment for age, gender, hypertension, diabetes, metastatic cancer, number of chronic illness, life-threatening event, life-time suicidal ideation, and depression. SRH may be a useful research tool to model medical service use for inpatients with chronic conditions.

## INTRODUCTION

Self-rated health (SRH), as a single-item measure, has been demonstrated to capture multiple dimensions of health and well-being.^1^ SRH has the potential to predict future health, functional loss related to well-being,^2,3^ and health care costs in western societies.^4,5^ Population-based studies have shown that SRH relates to the use of general health services and could predict hospital admissions.^3,4,6^ However, there is a paucity of studies on SRH and service use in tertiary care settings (ie, primary care hospitals) in Asian societies such as Taiwan. Furthermore, it has been noted that a more informative approach to the question of whether SRH influences hospitalizations takes into account repeated hospitalizations.^7^ To our knowledge, the impact of SRH on repeated hospitalizations in Taiwan has not been modeled with respect to hospitalized patients with preexisting chronic medical conditions.

Studies about psychosocial factors and hospital service use (ie, outpatient service, emergency department, and hospitalization) are limited globally.^8^ In Taiwan, studies have been limited to specific sub-populations that would not be defined on the basis of hospital-based common chronic medical conditions that can result in significant morbidity or eventual mortality. The ability of patients to self-select doctors and hospital services is frequent in Taiwan, and the concept of doctor-shopping has been established as well as more frequent use of emergency facilities.^9,10^ Reassurance seeking behaviors for patients’ health anxiety, which also relates to poor perception of health can influence repeated use of medical services.^11^ A better understanding of health-seeking in hospitalized patients with chronic disease as a consequence of poor perceived health may provide further clues to management strategies, resource allocation, and provide more accurate predictive models of health seeking in patients with preexisting chronic conditions.

The relationship between SRH and health service use is complex, especially within the hospital environment. In a cross-sectional study of hospitalized elderly, perceived health depended on severity of co-morbid illness and coping skills (resilience).^12^ Barsky et al^13^ has explained patient's medical morbidity, psychiatric co-morbidity, and functional disability as factors that may influence subjective ratings of SRH. Patients with mental health problems are likely to have poor affect SRH^14^ and likely to be more frequent users of primary care services that in turn impact inpatient admissions.^15^ Specifically, depression, somatization, anxiety, and life threatening events tend to reduce subjective well-being and are related to more use of health services.^6,16–18^ Further, Pu et al^10^ showed that the association between depressive symptoms and outpatient visits could be modulated by SRH. Therefore, a more rigorous approach to understand the relationship between SRH and health service for medical inpatients is to adjust for socio-demographic factors, medical comorbid condition, and psychosocial factors in a model.

Our aim was to investigate factors associated with poor self-rated heath and model the association between poor SRH and subsequent utilization of hospital associated medical outpatient departments, emergency department visits, and medical ward admissions in medical inpatients with chronic conditions in Taiwan. We also controlled for patients medical conditions and psychological status that are potential confounders.

## METHODS

### Study Context and Procedures

The cross-sectional study was conducted in a 2000-bed academic medical center during November 2012 to 2013 in northern Taiwan. Such tertiary hospital has been previously documented multiple health-seeking behaviors for patients across different specialties and services, particularly with respect to ambulatory care utilization.^19^ The ethical approval was acquired from the study hospital (reference number: 201112091RIC).

Minimum sample size required to detect reliably a twofold increase in service use a sample size of 160 patients is needed (alpha = 0.05; beta = 0.80) for a 20% base rate of frequent service use. The sample-size was boosted to 230 to control for potential confounding factors. Consecutive medical inpatients with various physical chronic illnesses admitted through the emergency department were recruited to the study. All eligible inpatients who admitted to the medical departments were provided full instructions and written informed consent before they were interviewed. Exclusion criteria included patients with moderate to severe cognitive dysfunction assessed by the SPMSQ (Short Portable Mental State Questionnaire) with education taken into consideration, for example, wrong answer ≤3 items for those with education years ≥12 was considered mild cognitive impairment.^20^ All patients were interviewed by a trained research assistant at bedside for 30 to 40 min using a standardized questionnaire.

### Measurements

#### Dependent Variables

##### Self-Rated Health

The participants were asked about their health via a single question: “In general terms, how would you describe your overall health status: very good, good, fair, poor, or very poor?” To ensure reliability, no health-related questions that could interfere or influence the response of patients were allowed before this, such as current medical conditions.

##### Medical Service Utilization

The frequency of medical service utilization was recorded as number of visits to medical outpatient services, emergency services, and hospitalization services in the past 12 months. The information was obtained from the patient's medical records as well as self-report frequencies of medical visits that added to previous records. For the purpose of analyses frequent users of outpatient service was defined as those patients in the upper tertile of outpatient service use in the previous year. Frequent users of emergency service were patients who visited emergency department 2 or more times in the previous year. Likewise frequent users of hospitalization services are the patients who have been admitted 2 or more times in the previous year.

#### Independent Variables

##### Socio-Demographic Variables

Age, gender, education years, and the status of living alone were collected. The subjects’ past and current medical and physical conditions were defined based on hospital diagnoses from the electronic medical records.

##### Medical Illnesses

This information was obtained from the patients’ electronic medical records. The Charlson comorbidity index was applied to assess the number and severity of comorbid conditions of the index admission.^21^ We recorded each medical illness with International Statistical Classification of Diseases and Related Health Problems (ICD) codes that encompass cancer and major organ systems such as brain, heart, liver, kidney, and the blood system.

##### Depressive Symptoms

The Patient Health Questionnaire-9 (PHQ-9) was used to assess the severity of depressive symptoms. The 9-item scale is rated on a 4-point scale, ranging from 0 (never) to 3 (nearly every day) generating a total score ranging from 0 to 27. Higher scores indicate increased likelihood of depressive disorders. The Chinese version of the PHQ-9 has been validated to have good reliability and validity in hospital and primary care patients in Taiwan.^22^

##### Threatening Life Events

This variable is defined as significant events in the patients’ life that can be stressful or life-threatening. Twelve such events occurred in the past 1 year were assessed using the list of Life-threatening Event Questionnaire (LTE-Q).^23^ These events fall into several categories including severe physical problems or death, legal problems, serious financial concerns, and other stressful events occurring to the family.

##### Life-Time Suicide Ideation

We assessed the patients’ life-time suicide ideation as an indicator of suicide risk. Other risk factors of suicide such as excessive ethanol or drug use, rational thinking loss, poor social support were assessed based on a brief rating scale, the Chinese SAD PERSONS Scale.^24^

### Data Analyses

Preanalysis included univariate and bivariate tabulation as a prerequisite for multivariate analyses. Associations between independent variables and poor SRH or frequent medical service utilization were performed using chi-squared tests and then were presented as odds ratios (ORs) with their 95% confidence intervals (CIs). Multivariate logistic regression was applied to investigate the independent effect of SRH on medical service utilization after separate and successive inclusion of other independent variables in the model. The model was adjusted for age, gender, life-threatening events in the past year, life-time suicide ideation, hypertension, diabetes, metastatic cancer, and number of chronic illness. All statistical analyses were performed using the SPSS v21, and statistical significance was defined at a value of *P* < 0.05.

## RESULTS

We obtained information on SRH in 230 consecutive inpatients at the medical departments in a medical center in northern Taiwan. The mean (standard deviation, SD) age was 62.6 (15.9) years and 46.5% were women. Patients who were not included in this study were somewhat older (mean ages 76 years), and the reasons of nonparticipation involved early discharge (n = 35), being excluded due to conscious disturbance or unable to be interviewed (n = 46) and refusal (n = 4), being isolated due to infection (n = 1), or transfer to other wards (n = 2). The average number of years of education was 9.3 (4.7) years, and 7% of patients had no formal education. Overall health was rated as very poor, poor, normal, good, and very good by 23.5%, 28.7%, 35.3%, 10%, and 2.6%, respectively. The mean number of chronic illnesses were 2.2 (range 0–7, SD = 1.4), with hypertension (46.1%), diabetes (24.3%), peptic ulcer disease (18.7%), chronic kidney disease (20%), metastatic cancer (17.4%), and congestive heart failure (14.8%) as the most common chronic disease diagnoses. Among the psychological factors examined 29.1% had life-time suicide ideation, 35% reported experiencing life-threatening events in the previous year, and 30% had moderate to severe depression using the PHQ-9. The mean number of visits to the medical outpatient service was 11.8 (13.1), emergency department was 1.2 (1.8), and hospital admissions was 1.0 (2.0).

### Factors Associated With Poor SRH

Socio-demographic information of the patients is presented in Table 1. Gender, age, education, and living status were not associated with poor SRH in this study (Table 2). The psychological factors significantly related to poor SRH included the presence of life-threatening events (OR = 1.7 (95% CI = 1.1–3.0)), life-time suicide ideation (OR = 2.9 [95% CI = 1.6–5.3]), and depression (OR = 3.8 [95% CI = 2.0–7.0]). Metastatic cancer was the only chronic medical condition associated with higher odds of poor SRH (OR = 3.3 [95% CI = 1.5–7.2]).

**TABLE 1:**
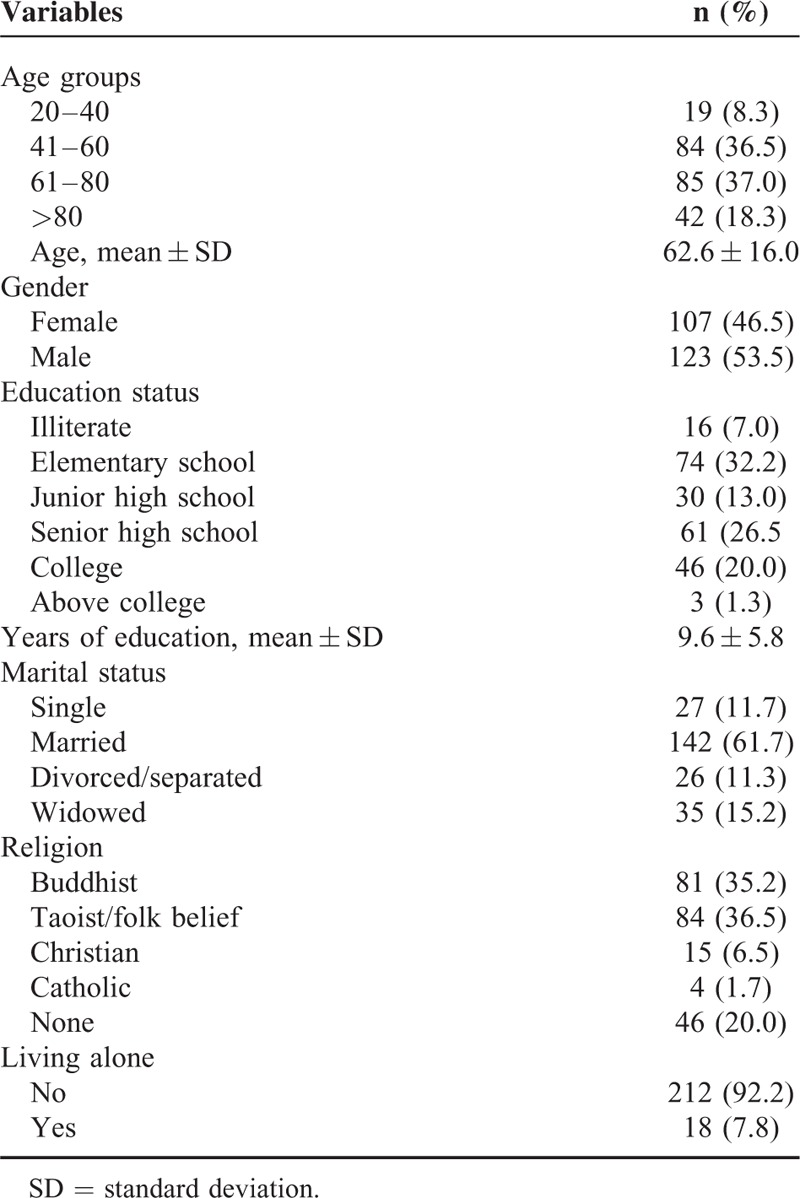
Socio-Demographic Information of the Study Participants (N = 230)

**TABLE 2:**
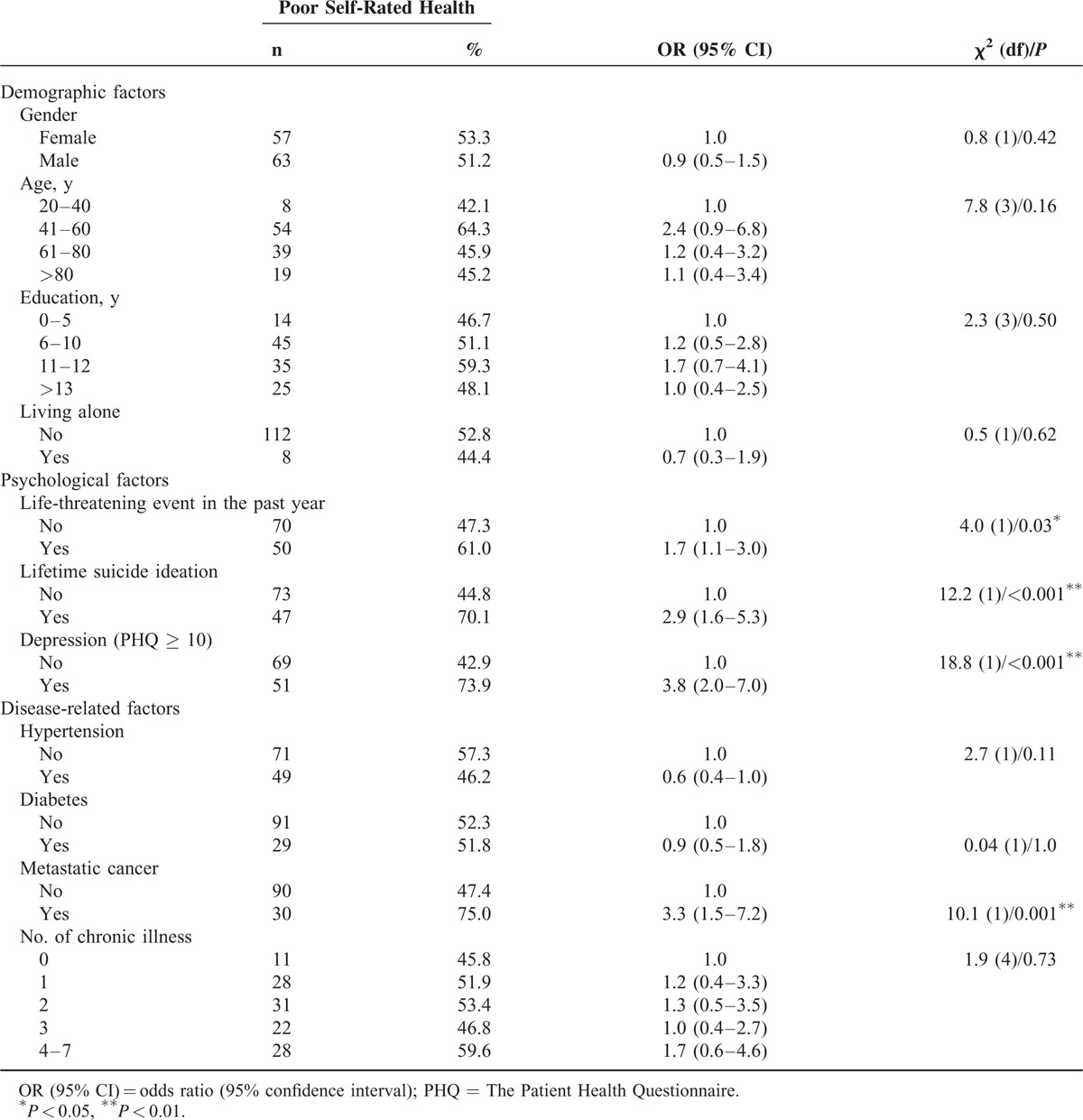
Factors Associated With Poor Self-Rated Health

### Factors Associated With Frequent Health Service Utilization

Table 3  displays the factors associated with frequent use of medical services, including medical outpatient services, emergency services, and hospitalization services. Men were less likely to use hospitalization services compared to women (OR = 0.4 [95% CI = 0.2–0.8]), and a similar but insignificant trend was seen in medical outpatient use. In our sample, no association between age and medical service use was seen.

**TABLE 3:**
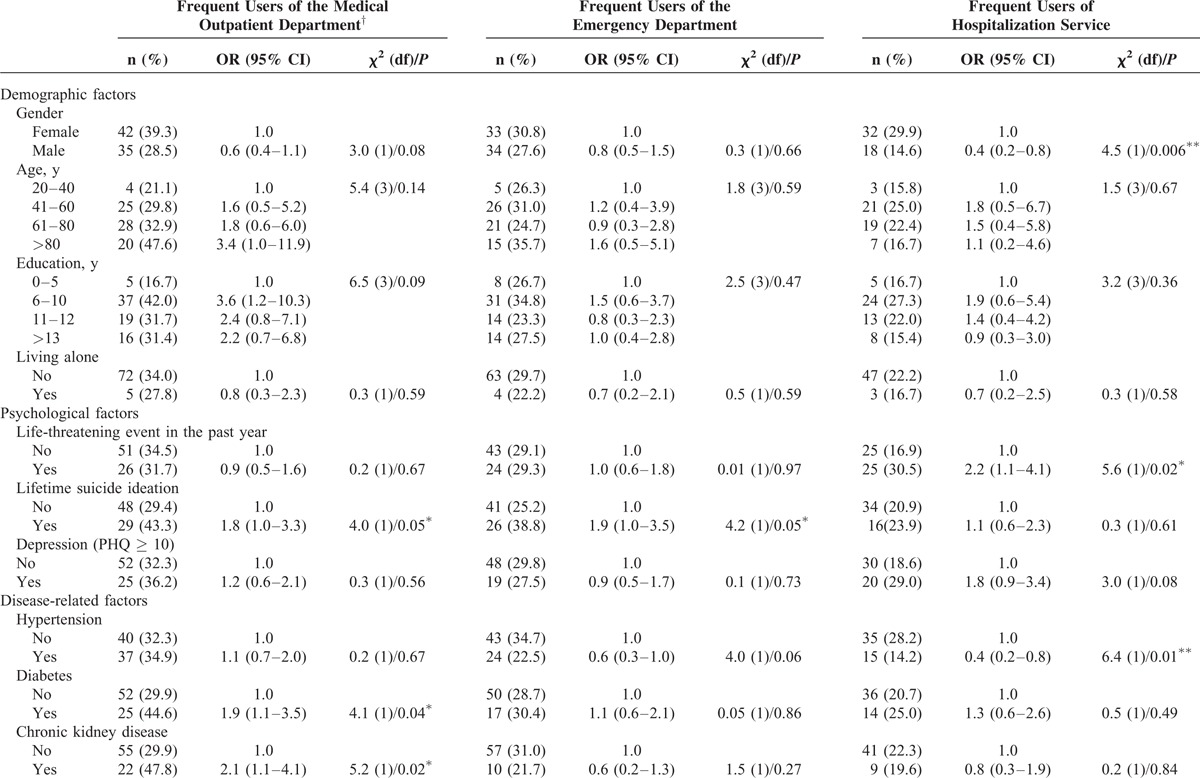
Factors Associated With Medical Service Utilization in the Previous Year

**TABLE 3 (Continued):**
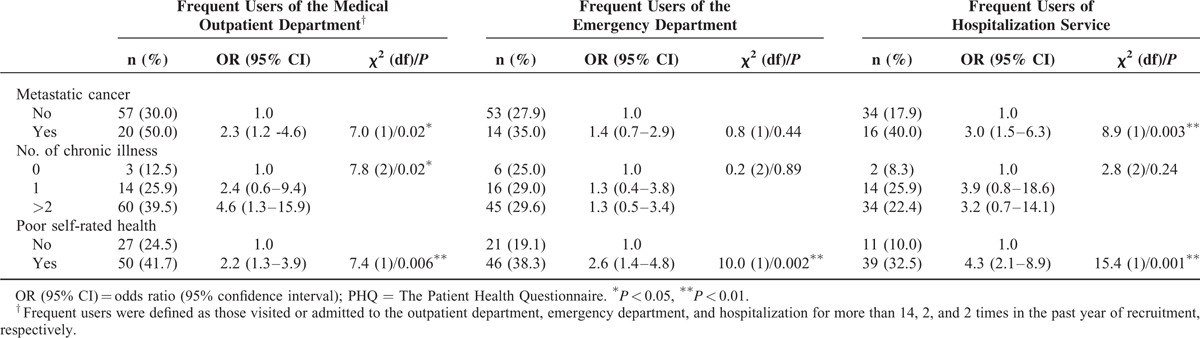
Factors Associated With Medical Service Utilization in the Previous Year

Chronic disease-related factors had an interactive influence on the frequency of medical service use. Higher odds of seeking medical outpatient services were seen in patients with diabetes (OR = 1.9 [95% CI = 1.1–3.5]), chronic kidney disease (OR = 2.1 [95% CI = 1.1–4.1]), and metastatic cancer (OR = 2.5 [95% CI = 1.2–5.0]). Patients with 2 or more chronic illnesses more frequently used medical outpatient service (OR = 2.3 [95% CI = 1.2–4.3]). On the contrary, patients with hypertension were less likely to use hospitalization services compared to patients without hypertension (OR = 0.4 [95% CI = 0.2–0.8]).

Among the psychological factors life-time suicide ideation was related to frequent use of medical outpatient services (OR = 1.8 [95% CI = 1.0–3.3]) and emergency services (OR = 1.9 [95% CI = 1.0–3.5]). Life-threatening events in the previous year were related to frequent use of hospitalization services (OR = 2.2 [95% CI = 1.1–4.1]). Depressed patients had higher odds of using hospitalization services although not statistically significant (OR = 1.8 [95% CI = 0.9–3.4]).

### Association Between Poor SRH and Health Service Utilization

In unadjusted analyses (Table 3 ), Poor SRH was associated with frequent use of medical outpatient services (OR = 2.2 [95% CI = 1.3–3.9]), emergency services (OR = 2.6 [95% CI = 1.4–4.8]), and hospitalization services (OR = 4.3 [95% CI = 2.1–8.9]). Logistic regression analyses were carried out to investigate confounding issues for the association of interest (Table 4). The OR for the fully adjusted association between poor SRH and medical outpatient services was OR = 2.0 (95% CI = 1.1–3.9); emergency services (OR = 2.8 [95% CI = 1.4–5.4]), and hospitalization (OR = 4.3 [95% CI = 1.9–10.0]). The model included age, gender, life-threatening event, life-time suicidal ideation, hypertension, diabetes, metastatic cancer, and number of chronic illness (Table 4).

**TABLE 4:**
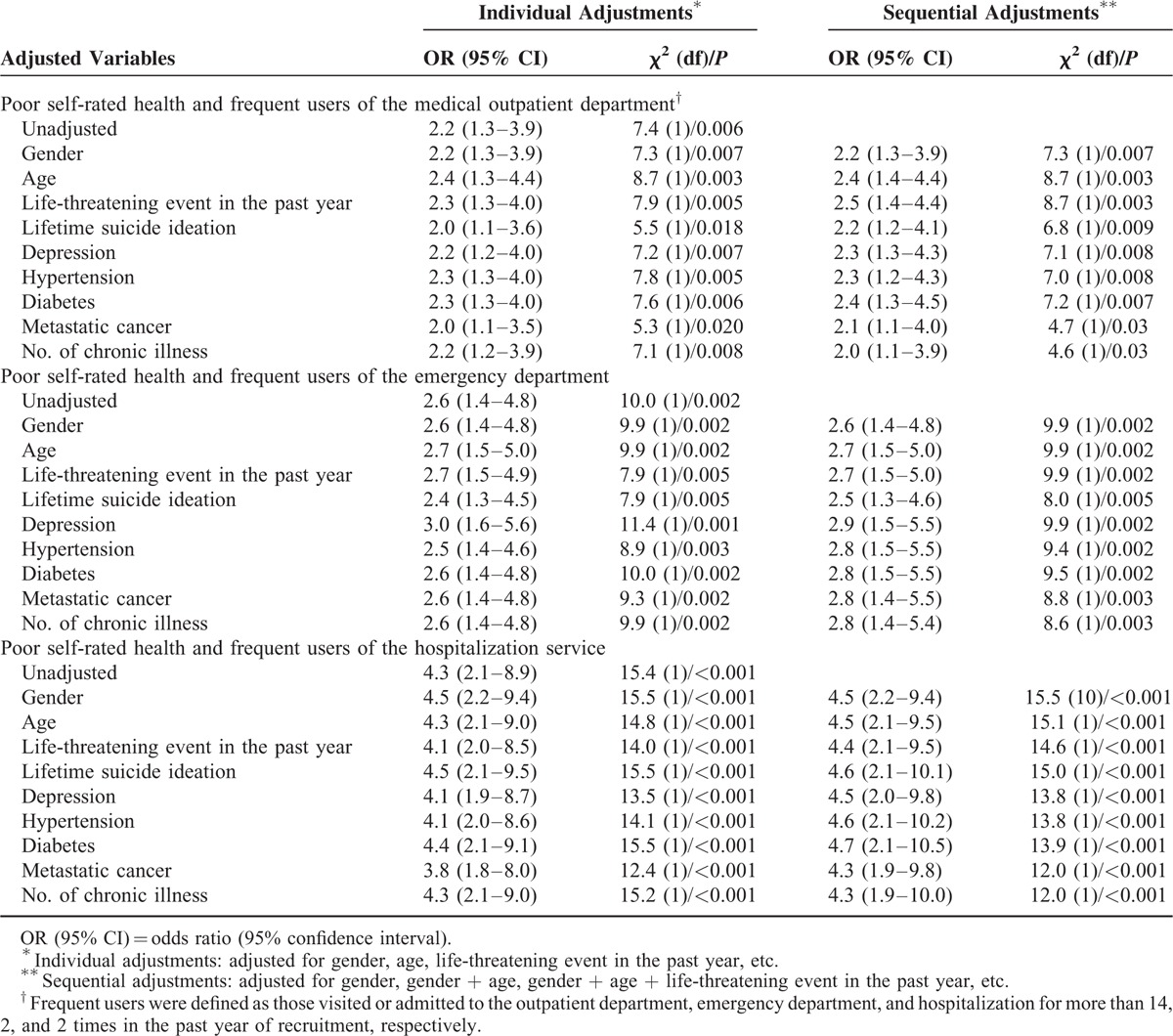
Multivariate Logistic Regression Analyses for the Association Between Poor Self-Rated Health and High-Frequency Service Use at Medical Outpatient Department, Emergency Department, and Hospitalization

## DISCUSSION

In a consecutive cohort of medical inpatients, we demonstrated that poor SRH was associated with frequent visits to the hospital medical outpatient clinics, emergency department, or medical in-hospital services. Patients with depression, life-time suicide ideation, life-threatening events in the past year and metastatic cancer were more likely to have poor SRH. Diabetes, chronic kidney disease, and metastatic cancer were related to frequent use of outpatient services. Additionally, suicide ideation and life events were associated with frequent use of outpatient services and hospital admissions, respectively. However, the increased use of hospital services with poor SRH was independent of our patient cohort's medical conditions or mental health.

While there is universal health care coverage in Taiwan, diabetes remains diagnosed latter in the disease cycle and poorly controlled in lower socio-economic groups.^25^ This may explain in part a higher rate of readmissions in diabetic patients in our cohort where the complications of under controlled diabetes could have impacted on health services. On the other hand, diabetes and SRH did not show a significant interaction that predicted significant increased hospital utilization. Patients may be unaware of their diabetic status or may have low level of health literacy and not have understood fully the impact of their own condition. Previous studies have shown that people with undiagnosed hypothyroidism, diabetes mellitus, or hypertension often have good SRH; however, once they were diagnosed with these diseases, they would report reduced SRH.^26^

Population-based studies have shown significant association between medical or psychological morbidity and poor SRH^6,14^; however, few of them specifically enrolled hospital inpatients. A search of keywords “self-rated health” and “Taiwan” in PubMed yielded over 100 publications in the last 20 years, but none have explored hospital re-admissions in an inpatient population in a general medical ward. It was found that medical status, functional status in daily living, and psychiatric morbidity have an adverse effect on patient's SRH.^12^ In a random sample of general medical clinic patients, health anxiety and somatization but not medical status were related to perceived global health.^13^ In our study, we showed that depression, recent life-threatening events, and lifetime suicide ideation were associated with poor SRH. Interestingly, we noted other than metastatic cancer no other chronic co-morbid conditions were associated poor SRH. Cancer survivors experience significant psychosocial morbidity and are influenced by several intrapersonal and environmental factors that affect their subjective perception of health.^27^ SRH has been found to be an independent predictor of survival in advanced cancer patients.^28^

In terms of hospital service utilization, our results are consistent with the limited studies on medical inpatients with chronic conditions. One study on adult inpatients reported patients with mental disorders utilized more health services than patients without mental disorders.^15^ Another study demonstrated depression, somatization, and the presence of life-threatening physical diseases were related to higher use of inpatient admissions. Our results demonstrate life-time suicide ideation is associated with frequent visits to both outpatient and emergency department. It could be possible that suicidal ideation could be the underlying cause of frequent medical service use; however, this assumption needs to be tested if the suicidal behavior or self-harm history resulted in higher risk of mortality^29^ or service utilization.

Frequent use of emergency services has been linked to physical and mental health.^30^ We also showed life-threatening events in the previous year were associated with frequent use of emergency service. Depression is a common factor on poor SRH and medical service use. Several studies have shown that depressive symptoms predict recurrent admission among patients with chronic medical condition.^31,32^ We showed that depression was strongly associated with poor SRH but was a weaker association with frequent hospitalization. It is possible that SRH eliminates the positive relationship between depressive symptoms and medical service use.^10^

The salient feature of our results is the consistency of association between poor SRH and frequent use of different medical services, that is, medical outpatient service, emergency department, and hospital admissions. A national survey of mental health and well-being in Australia showed SRH was associated with higher rates of being hospitalized over-night even after adjusting for psychiatric and physical morbidity.^6^ Among hospital services, Hansen et al^15^ showed SRH as a function of disability was associated with higher use of primary care services but not inpatient admissions. Molarius and Janson^33^ have identified that while many chronic diseases are strongly associated with poor SRH in the individual, common symptoms such as depression contribute more to the total burden of poor SHR in populations with chronic health problems. In our study, we choose to focus on SRH as opposed to SRH as a function of disability; however, we have captured important modifying factors such as depression. We acknowledge that some of chronic health condition types in our cohort would have impacted on disability and disability would have increased overtime. However, there is growing appreciation that SRH is a broad construct related to the self-concept of health, rather than reflecting the actual medical health status.^34^ We note previous studies have established that a single measure of a single measurement of SRH also predicts long-term patterns of hospitalization, for example, in heart failure, among older adults.^7^

In an effort to understand the direction of this relationship between SRH and medical service utilization, we were able to show an independent association between poor SRH and frequent use of various hospital services even after controlling for chronic conditions and psychological status. Other studies have suggested a long length of follow-up for assessment of hospital service use. In this study, we chose 1 year as the follow-up period in order to adequately model health service use in a chronically diseased population. Our study is limited by retrospective analysis and hence a reduced ability to determine the direction of causality. On the other hand, we found SRH was useful in predicting hospital service utilization and is amenable to retrospective analysis.

We explored a number of covariate factors including depression and low health literacy. These factors were explored to determine if these also impacted on health seeking behaviors and interacted with SRH. The association between psychological morbidities and poor SRH may suggest the need for psycho-social supportive service among medical inpatients. Low health literacy has been associated with frequent attendance to medical services in previous studies; although a study from Taiwan found no association between low health literacy and health service use.^9^ In the context of Taiwan, our study is of paramount importance as there is a growing need to be aware of likely re-hospitalizations and appropriate allocation of hospital resources. There is also a need to predict medical expenses in response to patients’ help-seeking or health-seeking behavior. SRH could be a simple indicator and an effective marker to help model medical service utilization, plan hospital resources allocation, and model medical expenses.
